# A Severe Case of *Plasmodium falciparum* Malaria in a 44-Year-Old Caucasian Woman on Return to Western Romania from a Visit to Nigeria

**DOI:** 10.3390/life14111454

**Published:** 2024-11-09

**Authors:** Alin Gabriel Mihu, Rodica Lighezan, Daniela Adriana Oatis, Ovidiu Alexandru Mederle, Cristina Petrine-Mocanu, Cristina Petrescu, Mirandolina Eugenia Prisca, Laura Andreea Ghenciu, Cecilia Roberta Avram, Maria Alina Lupu, Adelaida Bica, Tudor Rareș Olariu

**Affiliations:** 1 “Aurel Ardelean” Institute of Life Sciences, Vasile Goldis Western University of Arad, 310414 Arad, Romania; alinmg@yahoo.com; 2 Department of Biology and Life Sciences, Vasile Goldis Western University, 310300 Arad, Romania; 3 Center for Diagnosis and Study of Parasitic Diseases, Department of Infectious Disease, Victor Babes University of Medicine and Pharmacy, 300041 Timisoara, Romania; lighezan.rodica@umft.ro (R.L.); lupu.alina@umft.ro (M.A.L.); rolariu@umft.ro (T.R.O.); 4 Discipline of Parasitology, Department of Infectious Diseases, Victor Babes University of Medicine and Pharmacy, 300041 Timisoara, Romania; 5 Regional Blood Transfusion Center, 300737 Timisoara, Romania; 6 Patogen Preventia, 300124 Timisoara, Romania; 7 Department of Infectious Diseases, Arad Clinical Emergency Hospital, 310031 Arad, Romania; dr_prisca@yahoo.com (M.E.P.); adelabica@yahoo.com (A.B.); 8 Department of Medicine, Vasile Goldis Western University, 310300 Arad, Romania; 9 Surgery Department, Faculty of Medicine, Victor Babes University of Medicine and Pharmacy, 300041 Timisoara, Romania; mederle.ovidiu@umft.ro; 10 Emergency Department, Emergency Clinical Municipal Hospital, 300079 Timisoara, Romania; 11 Clinical Laboratory, Arad Clinical Emergency Hospital, 310031 Arad, Romania; petrinecristina@gmail.com; 12 Discipline of Hygiene, Department of Microbiology, Victor Babes University of Medicine and Pharmacy, 300041 Timisoara, Romania; petrescu.cristina@umft.ro; 13 Department of Functional Sciences, Victor Babes University of Medicine and Pharmacy Timisoara, 300041 Timisoara, Romania; bolintineanu.laura@umft.ro; 14 Center for Translational Research and Systems Medicine, Victor Babes University of Medicine and Pharmacy Timisoara, 300041 Timisoara, Romania; 15 Clinical Laboratory, Institute of Cardiovascular Diseases, 300310 Timisoara, Romania; 16 Clinical Laboratory, Municipal Clinical Emergency Teaching Hospital, 300254 Timisoara, Romania

**Keywords:** *Plasmodium falciparum*, malaria, fatal malaria case, Romania

## Abstract

Malaria is currently the most prevalent life-threatening infectious disease in the world. In this case report, we present a 44-year-old Caucasian woman with a low level of education and no significant past medical history who presented to the emergency room of the Emergency County Hospital of Arad, Romania, with a general affected state, a fever of 38.5 °C, chills, weakness, headache, muscle pain, nausea, icterus, and watery diarrheal stool. A viral infection was initially suspected, and the patient was transferred to the Infectious Diseases Department. The anamnesis revealed that the patient traveled to Nigeria (Ado Ekiti) and returned to Romania 14 days before presenting to the hospital without following antimalarial prophylaxis. A peripheral blood smear was conducted and revealed parasitemia with ring forms of *Plasmodium falciparum* (*P. falciparum*) of 10–15% within the red blood cells. Parasitemia increased within a day to 15–18%, and her health rapidly deteriorated. She was transferred to the Victor Babeș Infectious Disease Hospital in Bucharest for the urgent initiation of antimalarial treatment. The patient’s condition continued to worsen rapidly, and she succumbed to her illness due to multi-organ failure. This report details the first documented case of malaria imported from Nigeria to Romania. People traveling to malaria-endemic areas should be educated about preventing this parasitic infection, both by adopting measures to reduce the risk of mosquito bites and by using appropriate chemoprophylaxis. In the context of resuming travel after the COVID-19 pandemic, understanding and adhering to prophylactic measures is crucial to avoid tragic situations, as highlighted in this case report.

## 1. Introduction

Malaria remains the single most prevalent life-threatening infectious disease in the world [1]. This parasitic infection is endemic in over 90 countries and affects approximately 40% of the global population [2]. According to the latest World malaria report, there were 249 million cases of malaria in 2022 compared to 244 million cases in 2021 [1].

*Plasmodium falciparum *(*P. falciparum*) and *Plasmodium vivax *(*P. vivax*) are the main species that cause malaria infection in humans [3]. Malaria caused by *P. falciparum* is the most severe form of disease, and it is accompanied by the highest number of deaths worldwide compared to infections caused by other species of the parasite [4]. Infection with *P. falciparum* can be fatal if prompt diagnosis and appropriate treatment are not initiated [5]. The vector for *Plasmodium* spp. is represented by the female mosquito of the genus *Anopheles*, which injects sporozoites present in its salivary glands during the bite [6]. The extrinsic incubation period in malaria represents the time interval between when a mosquito is infected with the malaria parasite and when it can transmit the infection through its bite. Typically, this period ranges from 8 to 14 days [7]. The intrinsic incubation period in this parasitic infection is the interval of time between when a person is bitten by an infected mosquito, which introduces the parasite into the human host’s bloodstream, and when the symptoms of the disease become evident. This period can vary between 7 and 30 days [8].

The symptoms of uncomplicated malaria are generally nonspecific and include fever, cough, chills, myalgia, headache, and anorexia [9,10]. Occasionally, patients present with gastrointestinal symptoms, respiratory symptoms, and jaundice [11]. The clinical manifestations of severe *P. falciparum* infection usually appear after 3 to 7 days of the initial symptoms [2]. These include respiratory distress (acidotic breathing), prostration, and impaired consciousness [9]. Other symptoms of severe malaria include multiple seizures, radiologically confirmed pulmonary edema (respiratory failure due to acute lung injury progressing to acute respiratory distress syndrome), abnormal bleeding (disseminated intravascular coagulation), acute kidney injury, jaundice, shock, and coma [9,10]. Laboratory characteristics in severe malaria may indicate severe anemia, hypoglycemia, acidosis, hyperlactatemia, hyperparasitemia, and renal failure [2].

Malaria can be diagnosed using rapid diagnostic tests (RDTs) [12], peripheral blood smears, which are a simple and rapid technique [13], or polymerase chain reaction (PCR)-based assays [14]. RDTs should be followed by confirmation through microscopy, and in the case of a positive result, the quantification of parasitemia should be performed [5]. PCR techniques can have a sensitivity 100 times greater compared to RDTs [15,16,17], especially in patients with low parasitemia [18] or those with subclinical infections [19].

Currently, the microscopic method is the gold standard for the detection and diagnosis of malaria [20]. The microscopic examination of thick and thin blood smears using Giemsa staining is the most common technique for the accurate identification of *Plasmodium* species [21]. Thick smears are generally more sensitive than thin smears in malaria for detecting low-density parasitemia [22]. Giemsa staining allows for a detailed observation of the morphological characteristics of parasites in infected blood [21]. If clinical suspicion is significant and the parasite is not detectable in the first blood smear, it is recommended to repeat the smear every 12–24 h for a total of three sets [23]. This approach increases the chances of detecting the parasite, as parasite density may be higher at certain times in the parasite replication cycle. If all three sets of blood smears are negative, the diagnosis of malaria can be excluded. Identifying the species of parasite causing the infection is essential for establishing the appropriate treatment protocol [21]. The rapid and accurate diagnosis of this infection combined with the prompt administration of appropriate antimalarial treatment are essential for reducing the complications and mortality associated with this infection [24].

The primary goal in treating malaria is the swift and complete elimination of *Plasmodium* parasites from the patient’s bloodstream to prevent disease progression. For uncomplicated malaria, the World Health Organization (WHO) recommends artemisinin-based combination therapies as the first-line treatment in endemic areas, particularly against *P. falciparum*. In cases of severe malaria, intravenous artesunate is the preferred treatment due to its rapid efficacy. This protocol helps not only in reducing patient mortality but also in preventing the transmission and emergence of drug resistance [25,26]. Hydroxychloroquine has historically been effective against certain non-resistant malaria strains, but is less suitable for *P. falciparum*, particularly in cases involving resistance. While hydroxychloroquine remains viable in select regions, artemisinin-based combination therapies are the recommended first-line treatment for *P. falciparum* malaria [26,27]. Drug-resistant malaria was reported to be an increasing global concern, especially with strains of *P. falciparum,* developing resistance to key treatments like artemisinin-based combination therapies. Resistance complicates malaria control efforts by reducing treatment effectiveness and prolonging infection duration, which can enhance transmission risk and the severity of the disease [28,29].

In Romania, malaria was eradicated in 1963 [30]. In 1948, Romania reported a total of 333.198 cases of malaria, but starting from 1968, no local transmission of malaria has been reported [31]. From 1980 to 2007, an average of approximately 20 imported cases were identified annually [32]. The diagnosed malaria cases in Romania were of patients returning from malaria-endemic areas, mainly from Africa [31,33,34]. The purpose of this report study was to present a new case of imported malaria detected in the city of Arad, western Romania.

## 2. Case Presentation

A 44-year-old Caucasian woman with a low level of education and no significant past medical history presented to the emergency room (ER) of the Emergency County Hospital of Arad, Romania, with a general affected state, a fever of 38.5 °C, chills, weakness, headache, muscle pain, nausea, icterus, and watery diarrheal stools. The symptoms began 7–10 days prior to hospitalization. The patient initially thought it to be a viral infection, most probably COVID-19 due to the wave of SARS-CoV-2 infections in Romania at that time. The symptoms slowly progressed, icterus appeared, and finally, she decided to seek medical attention.

Upon presentation in the ER, the patient was generally affected but conscious and cooperative, with a Glasgow Coma Scale (GCS) of 15 (motor response = 6, verbal response = 5, eye response = 4), a respiratory rate (RR) of 16 breaths/min, a blood pressure (BP) of 110/65 mmHg, a heart rate (HR) of 96 beats/min, and SpO_2_ of 98% in room air. The patient’s skin was warm and moist, with slight scleral icterus and dehydrated mucous membranes. A rectal exam and moderate metrorrhagia were noted. She was administered analgesics and antipyretics (Perfalgan, 1000 mg/100 mL and Metamizole, 1 g/2 mL) and 500 mL of 0.9% saline solution.

Results of the laboratory tests ordered in the ER are presented in Table 1. A chest radiography showed no significant changes. A COVID-19 rapid test was performed, and the result was negative. The patient was transferred to the Infectious Diseases Department with a suspicion of a viral infection for specialized investigations and treatment.

On transfer, the patient was generally affected but conscious and cooperative, hemodynamically and respiratory stable, with a GCS of 15, an RR of 17 breaths/min, a BP of 105/70 mmHg, an HR of 81 beats/min, SpO_2_ of 95% in room air, a temperature of 36.4 °C, slight scleral icterus, mild facial edema, dehydrated mucous membranes, moderate metrorrhagia, minimal rectal bleeding, accelerated intestinal transit, abdomen tender on diffuse palpation, and no signs of meningeal irritation.

The anamnesis conducted in the Infectious Disease Department revealed that the patient traveled to Nigeria (Ado Ekiti) and returned 14 days before presenting to the ER without following antimalarial prophylaxis but was vaccinated against yellow fever. She was the only family member showing symptoms. The patient’s travel history spanned 7 days, and she experienced chills without fever one day after returning to Romania, which subsided after self-medicating with non-steroidal anti-inflammatory drugs.

**Figure 1:**
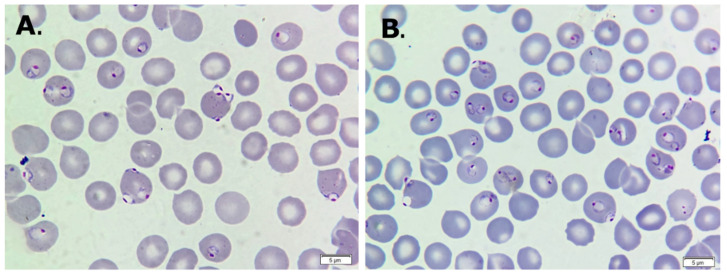
(**A**,**B**) May–Grunwald Giemsa-stained thin peripheral blood smear showing severe parasitemia with several ring forms of *Plasmodium falciparum *(×1000).

Given the epidemiological context and clinical signs, malaria was suspected, and a peripheral blood smear was quickly performed, revealing significant parasitemia with ring forms of *P. falciparum*. The parasitemia index was around 10–15% and was determined as previously described [35,36]. Therefore, a suspicion of malaria was raised (Figure 1A,B). The diagnosis was confirmed at the Center for Diagnosis and Study of Parasitic Diseases, Department of Infectious Disease from Victor Babes University of Medicine and Pharmacy in Timisoara, Romania.

Due to the unavailability of antimalarial treatment in the County Hospital of Arad, the patient was advised to be transferred to the Victor Babes Infectious Disease Hospital in Bucharest, the only clinical facility in Romania specialized in tropical diseases, including malaria. The patient declined the transfer and signed a refusal consent.

One day after the initial presentation to the ER, laboratory tests were repeated (Table 1).

**Figure 2:**
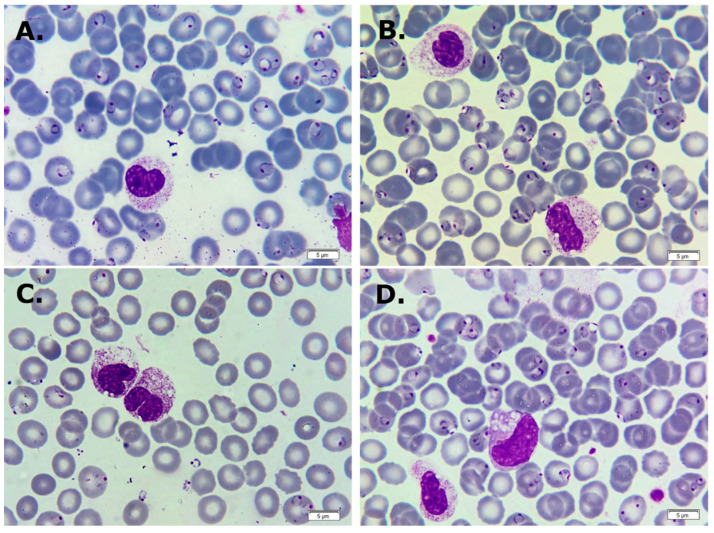
May–Grunwald Giemsa-stained thin peripheral blood smear showing severe parasitemia with ring forms of *Plasmodium falciparum*. (**A**) A band neutrophil with toxic vacuoles. (**B**) Two metamyelocytes with toxic granules and vacuoles. (**C**) Two band neutrophils with toxic granules and vacuoles. (**D**) A giant metamyelocyte with several vacuoles, toxic granules, and an unsegmented neutrophil with toxic granules (×1000).

A peripheral blood smear was conducted again and an increased parasitemia to 15–18% was noticed (Figure 2A). The leukocyte formula showed a marked left shift with 20% segmented neutrophils, 36% unsegmented neutrophils, 10% metamyelocytes, 8% myelocytes, toxic granules, and vacuoles that were noted in the granulocytes (Figure 2B).

**Figure 3:**
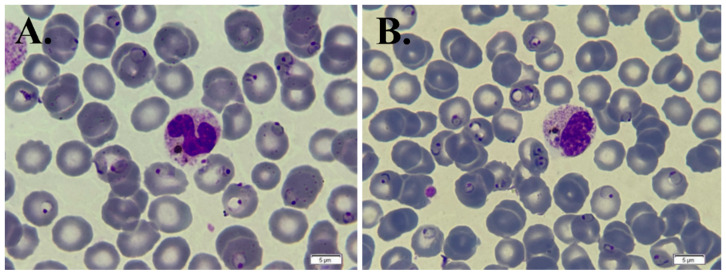
May–Grunwald Giemsa-stained thin peripheral blood smear showing severe parasitemia with ring forms of *Plasmodium falciparum* with an (**A**) unsegmented neutrophil and (**B**) metamyelocyte, both with the malaria pigment (hemozoin) (×1000).

Some granulocytes observed on the blood smear contained a crystalline brownish-black pigment, known as the malaria pigment, within their cytoplasm (Figure 3A,B).

The patient’s treatment during the two-day period is detailed in Table 2.

Due to her declining state of health, she finally agreed to be transferred by helicopter to the Victor Babes Infectious Disease Hospital in Bucharest, two days after the presentation to the ER, for the urgent initiation of antimalarial treatment. Unfortunately, the patient’s condition worsened rapidly, and death occurred shortly after arrival due to multi-organ failure.

## 3. Discussion

Malaria represents a major public health problem in sub-Saharan Africa, and Nigeria is one of the countries most affected by this parasitic disease [37]. Individuals traveling to sub-Saharan Africa have the highest risk of both contracting malaria and dying from this infection [5]. According to data provided by the WHO, Nigeria had a significant malaria burden in 2022, accounting for 27% of the total global malaria cases and 31% of the total malaria-related deaths worldwide [1].

Severe malaria, as defined by the World Health Organization (WHO), is a life-threatening condition caused by *P. falciparum*. It occurs when the infection leads to serious complications, often progressing rapidly from mild symptoms like fever, chills, and headaches to severe manifestations. These severe symptoms may include impaired consciousness, multiple convulsions, severe anemia, difficulty breathing, jaundice, and abnormal bleeding. Left untreated, severe malaria can result in organ failure and death, particularly within 24 h of symptom onset [38].

In Romania, imported cases of malaria have been reported [31,33], but none have originated from Nigeria. This is the first case of malaria reported in Arad (western Romania), which ultimately led to the death of the patient. The patient presented signs and symptoms of severe malaria, with multiple organ complications.

In 2011, a case of malaria with *P. vivax* was identified in a 25-year-old Romanian young man who contracted the infection following a trip to Greece [31]. In western Romania, during the period of 2010–2011, two cases of malaria infection were identified. The first case, caused by *P. vivax*, was recorded in a 20-year-old woman who traveled to India. The other reported case, of a 60-year-old woman, was caused by *P. falciparum* and was imported from Ghana [33]. In addition, between December 2023 and January 2024, four cases of malaria were reported in Romanian citizens who traveled to Zanzibar [34].

The symptoms of malaria can appear within a timeframe of 8–10 days or up to a year or even longer after a person has been infected with the parasite. Most travelers who develop malaria become ill within a few weeks or 1–2 months after returning home [5]. Fever is the most common symptom, and the presence of fever with or without other symptoms in a person who has returned from a malaria-endemic region requires testing to confirm or exclude the presence of infection, even if they have undergone chemoprophylaxis treatment [5].

Malaria caused by *P. falciparum* is considered the most severe form of the disease, as the parasite can infect and destroy many red blood cells in a short period of time. Infected red blood cells adhere to the walls of blood vessels in the microcirculation, which can lead to the blockage of blood flow and ischemia of the affected tissues [21]. In patients infected with malaria, laboratory test results may reveal the presence of normochromic/normocytic anemia, thrombocytopenia, leukopenia or leukocytosis, hyponatremia, hypoglycemia, and proteinuria. Additionally, this infection may be associated with increased markers of liver and kidney function and abnormal coagulation tests [39].

The hematological results of our patient showed the presence of anemia, thrombocytopenia, and elevated levels of D-dimer. Hematological abnormalities are a hallmark of malaria. These are more pronounced in *P. falciparum* infection, likely due to the higher level of parasitemia present in these patients [40]. Anemia is a frequent occurrence in malaria, and its pathogenesis is extremely complex. It is presumed to result from intravascular hemolysis and phagocytosis of infected red blood cells by the host’s immune system. If parasitemia is high, intravascular hemolysis can ultimately result in hemoglobinuria, and in severe cases, it may lead to renal failure [21]. Thrombocytopenia in our patient serves as a predictor of malaria. Thrombocytopenia is a common feature of malaria infection. A decreased platelet count has been observed in approximately 85% of patients with uncomplicated malaria and in all patients with severe forms of malaria caused by *P. falciparum* [40]. Thrombocytopenia was more common than anemia in acute malaria infection in patients with imported malaria [41,42]. The plasma level of D-dimer is an indicator of active thrombotic processes. Dasgupta et al. (2012) observed high levels of D-dimer in 87.5% of cases of malaria caused by *P. falciparum* and in 92% of patients infected with *P. vivax*, suggesting the presence of latent subclinical thrombosis [43].

Icterus is a complication frequently seen in severe malaria infection. It can be caused by intravascular hemolysis, disseminated intravascular coagulation, or malaria-related hepatic disorders [44]. Kochar et al. (2009) identified jaundice and liver dysfunction as among the most prevalent complications in severe *P. vivax* infection [45]. The increase in total and direct bilirubin values in our patient is the result of intravascular hemolysis. Additionally, hepatic dysfunction was noted. Infection with *Plasmodium* spp. significantly increased serum ALT and AST levels in malaria-diagnosed patients from Cameroon [46]. In a cohort study conducted in Ethiopia in 2020, high levels of AST, ALT, and ALP were reported in patients with this parasitic infection [47]. Pinto et al. (2019) noted the presence of severe thrombocytopenia and elevated levels of ALT, AST, LDH, GGT, and CRP in the case of a 28-year-old woman infected with *Plasmodium malariae* who traveled to Africa [48].

The reduced levels of sodium, potassium, and chloride suggest the presence of an electrolyte imbalance in the patient’s body, caused by excessive loss of fluids and electrolytes through diarrhea. According to the WHO, severe cases of malaria can be associated with electrolyte imbalances (sodium, potassium, chloride, calcium, and phosphate) that can worsen the patient’s condition [22].

In the case of our patient, the parasitemia was 10–15%, which represents an extremely high value, exceeding 5% being life-threatening [4]. The parasite index is used to estimate parasite density in the blood in cases of malaria infection. It represents the number of parasitized red blood cells per 100 red blood cells in a peripheral blood smear. Hyperparasitemia has been defined as a parasite index >5%. High parasitemia values are a marker of the severity of malaria infection [49]. The likelihood of a patient developing a severe form of the disease increases with higher parasite density in peripheral blood [22]. Patients infected with *P. falciparum* with hyperparasitemia have a higher risk of mortality [22,49]. High levels of parasitemia may indicate increased parasite resistance to antimalarial treatment, making them less susceptible to commonly used drugs [49]. Parasitemia >20% is associated with a high risk in any epidemiological context. Therefore, assessing prognosis in cases of malaria must take into account several factors, including the level of parasitemia and the local epidemiological context [22].

In this case, the presence of immature elements, vacuolated granulocytes, and toxic granulation was noteworthy. Immature neutrophils are usually found in the bone marrow but can also be detected in peripheral blood in infections and inflammation, including malaria [50,51]. The presence of immature neutrophils in peripheral blood can be an indicator of inflammatory activity and infection severity. Neutrophils may be involved in the development of complications associated with malaria by releasing toxic granules containing enzymes such as myeloperoxidase, neutrophil elastase, and matrix metalloproteinase-8 (MMP-8). This release can contribute to endothelial damage, causing endothelial cell apoptosis and subsequent edema [52].

The malaria pigment (hemozoin) is a crystalline brownish-black pigment produced by Plasmodium during hemoglobin digestion. Hemozoin accumulates in the parasite’s digestive vacuole to neutralize toxic byproducts (such as heme). Upon the rupture of infected red blood cells, hemozoin is released into the bloodstream, where immune cells like granulocytes and macrophages engulf it. Due to its resistance to degradation, hemozoin accumulates within these cells, triggering inflammatory responses central to malaria’s pathology [53,54,55].

The increase in inflammatory markers such as ferritin and CRP noted in this patient suggests the presence of severe infection. Elevated serum ferritin levels in patients diagnosed with malaria are an indicator of disease severity [56]. Elevated CRP levels are an important biomarker for the early detection of malaria infection and for monitoring disease severity [57], correlating with malaria parasitemia [58]. Patients who experienced multiple complications of malaria infection, as well as those who succumbed to the illness, exhibited higher CRP values compared to those who survived [59]. In our case, the presence of neutrophilia with a left shift and elevated levels of ferritin and CRP can indicate a strong inflammatory response in the body, which could suggest the presence of a severe infection, such as parasitic sepsis.

This case highlights the importance of raising awareness among travelers to malaria-endemic areas by healthcare professionals and authorities. Malaria prophylaxis is essential for individuals traveling to endemic zones, and informing and educating them can significantly reduce the risk of infection during their stay in these areas [60,61]. Infection prophylaxis involves avoiding mosquito bites by wearing appropriate clothing, using repellents or mosquito nets, and using specific chemoprophylaxis, which depends on the planned travel destination and the individual patient’s indications and contraindications. Chemoprophylaxis does not eliminate the risk of infection, but it significantly reduces it. If infection develops, it is milder and enhances the patient’s chances of recovery [62]. A large proportion of malaria infections, if not all, could be prevented with adapted prevention measures.

The CDC guidelines underscore the importance of rapid malaria treatment, particularly highlighting the need to treat malaria within 24–48 h to prevent complications, especially for *P. falciparum* cases [63]. Additionally, a review by Mousa et al. (2020) found that treatment delays significantly increase the risk of severe malaria, underscoring the need for hospitals, even in non-endemic regions, to stock essential antimalarial drugs for immediate intervention [64].

In Nigeria, the emergence of drug-resistant *P. falciparum* strains poses significant challenges to malaria treatment. Mutations in genes like *Pfcrt* and *Pfdhfr* have contributed to resistance against older antimalarial drugs, notably chloroquine and sulfadoxine–pyrimethamine, which once served as primary treatments [65,66]. These mutations were reported to be prevalent in Nigerian malaria isolates, reducing the efficacy of these drugs. Although artemisinin-based combination therapies have remained largely effective and are the recommended first-line treatment, certain studies have documented delayed parasite clearance times, which are associated with nonsynonymous mutations in the *PfK13* gene. While these mutations have not yet shown the same level of resistance as seen in southeast Asia, they suggest potential early stages of resistance in some Nigerian strains [67,68]. Surveillance studies on imported malaria cases further reveal that Nigerian isolates often carry these mutations, highlighting the need for continuous monitoring to adapt treatment strategies and sustain the efficacy of artemisinin-based combination therapies over time [68].

In individuals who have traveled to endemic areas, healthcare providers should always consider the possibility of malaria infection in febrile patients, regardless of symptoms. They should be promptly tested for malaria infection, as the early initiation of treatment is essential for reducing morbidity and mortality caused by malaria. Suspecting or confirming malaria, especially *P. falciparum* infection, represents a medical emergency that requires rapid intervention, as clinical deterioration can occur rapidly and unpredictably [5].

In areas where there are no indigenous cases of malaria, a travel history to endemic regions is essential for establishing a correct diagnosis in febrile subjects [9]. General practitioners should inquire whether patients presenting with fever or nonspecific signs have recently traveled to endemic areas to identify possible risks of malaria infection.

In the case history of our patient, several opportunities to avoid the fatal outcome were missed. First, she did not receive counseling from a travel medicine specialist and did not follow any type of chemoprophylaxis. Second, upon her return to Romania, she did not seek medical care and did not undergo antiparasitic treatment leading to complications and ultimately the patient’s death. The risk of developing severe malaria increases with a delay in initiating antimalarial treatment [69,70]. The longer the interval from symptom onset to diagnosis and treatment, the greater is the risk of death from this infection [71].

## 4. Conclusions

This report highlighted a case of severe malaria in a 44-year-old female who visited an endemic region in Africa without taking appropriate chemoprophylaxis. The current report is the first documented case of imported malaria from Ado Ekiti, Nigeria, to Romania. Given the rarity of malaria cases in Romania, especially those with potential drug-resistant strains, this case underscores the importance of heightened vigilance, prompt diagnosis, and access to effective antimalarial treatments. People traveling to malaria-endemic areas need to be educated about preventing this infection, both by adopting measures to reduce the risk of mosquito bites and by using appropriate chemoprophylaxis. Suspected cases of malaria should be tested immediately and should be treated as medical emergencies. As global travel resumes post-COVID-19 pandemic, understanding and adhering to preventive health measures are essential in order to prevent tragic health outcomes associated with infectious diseases, such as the malaria case documented in this report. Emphasizing the need for awareness and prevention strategies, this case serves as a reminder of the ongoing risks and the crucial role of preventive measures in safeguarding public health during international travel.

## Figures and Tables

**Table 1:** Laboratory results of the blood tests conducted on a 44-year-old Caucasian woman with *Plasmodium falciparum* malaria.

Laboratory Test	Normal Range	Day 1	Day 2
White Cell Count (WBC)	4000–11,000/µL	4020/µL	5620/µL
Neutrophils	40–60%	87.90%	72.40%
Lymphocytes	20–40%	8.20%	4.07%
Eosinophils	1–4%	1.50%	0.50%
Platelets	150,000–450,000/ÂµL	8000/ÂµL	16,000/µL
Hemoglobin	12.0–15.5 g/dL	14.70 g/dL	13.30 g/dL
Hematocrit	34.9–44.5%	42.70%	37.40%
Mean Corpuscular Volume (MCV)	80–100 fL	89.2 fL	89 fL
Blood Glucose	70–110 mg/dL	108.72 mg/dL	-
Blood Urea Nitrogen (BUN)	7–20 mg/dL	59.80 mg/dL	-
Creatinine	0.6–1.3 mg/dL	1.67 mg/dL	-
Estimated Glomerular Filtration Rate (eGFR)	>60 mL/min/1.73 m^2^	36.95 mL/min/1.73 m^2^	-
Alanine Transaminase (ALT)	7–56 U/L	179.3 U/L	-
Aspartate Transaminase (AST)	10–40 U/L	172.3 U/L	-
D-dimer	<0.5 µg/mL FEU	>5.00 µg/mL FEU	48.10 µg/mL FEU
Presepsin	60–400 pg/mL	3960 pg/mL	-
Amylase	23–85 U/L	18.2 U/L	-
Sodium	135–145 mmol/L	-	136 mmol/L
Potassium	3.5–5.1 mmol/L	-	3.53 mmol/L
Chloride	98–107 mmol/L	-	104 mmol/L
Lactate Dehydrogenase (LDH)	140–280 U/L	-	464 U/L
Total Bilirubin	0.1–1.2 mg/dL	-	6.22 mg/dL
Direct Bilirubin	0–0.3 mg/dL	-	5.58 mg/dL
Gamma-glutamyl Transferase (GGT)	9–48 U/L	-	107 U/L
Alkaline Phosphatase (ALP)	44–147 U/L	-	111 U/L
Interleukin-6 (IL-6)	<7 pg/mL	-	57.45 pg/mL
Procalcitonin	<0.05 ng/mL	-	6.04 ng/mL
Thrombin Time	12–14 s	-	66 s
Activated Partial Thromboplastin Time (APTT)	21–35 s	-	36.20 s
Ferritin	13–150 ng/mL	-	>2000 ng/mL
Total Protein	6.0–8.3 g/dL	-	5.73 g/dL
C-reactive Protein (CRP)	<10 mg/L	-	201.18 mg/L

**Table 2:** Initial medication administered to a 44-year-old Caucasian woman with *Plasmodium falciparum* malaria.

Medication	Dose
Fitomenadion	1 vial of 10 mg/mL
Etamsylate	1 vial of 250 mg/2 mL
Adrenostazin	1 vial of 0.3 mg/mL
Amoxiplus	2 vials of 1000 mg/200 mg
Aspatofort	2 vials of 10 mL
Arginine–Sorbitol	1 vial of 250 mL (50 mg/mL + 100 mg/mL)
Pantoprazole SUN	1 vial of 40 mg
Sodium Chloride 0.9%	2 vials × 500 mL
Glucose 5%	1 vial × 500 mL
Platelet Mass	4 units
Fresh Frozen Plasma (FFP)	1 unit

## Data Availability

Data are contained within the article.
